# Vehicle Instance Segmentation Polygonal Dataset for a Private Surveillance System

**DOI:** 10.3390/s23073642

**Published:** 2023-03-31

**Authors:** Najmath Ottakath, Somaya Al-Maadeed

**Affiliations:** Department of Computer Science and Engineering, Qatar University, Doha P.O. Box 2713, Qatar; s_alali@qu.edu.qa

**Keywords:** instance segmentation, classification, vehicle make classification, mosaic-tiled augmentation

## Abstract

Vehicle identification and re-identification is an essential tool for traffic surveillance. However, with cameras at every corner of the street, there is a requirement for private surveillance. Automated surveillance can be achieved through computer vision tasks such as segmentation of the vehicle, classification of the make and model of the vehicle and license plate detection. To achieve a unique representation of every vehicle on the road with just the region of interest extracted, instance segmentation is applied. With the frontal part of the vehicle segmented for privacy, the vehicle make is identified along with the license plate. To achieve this, a dataset is annotated with a polygonal bounding box of its frontal region and license plate localization. State-of-the-art methods, maskRCNN, is utilized to identify the best performing model. Further, data augmentation using multiple techniques is evaluated for better generalization of the dataset. The results showed improved classification as well as a high mAP for the dataset when compared to previous approaches on the same dataset. A classification accuracy of 99.2% was obtained and segmentation was achieved with a high mAP of 99.67%. Data augmentation approaches were employed to balance and generalize the dataset of which the mosaic-tiled approach produced higher accuracy.

## 1. Introduction

Vehicle surveillance is an essential task in public security [1]. Unique features of vehicles such as the make, model, and license plate are typically utilized for traffic surveillance [2]. With traffic cameras at every intersection, the entrances of high-security buildings, parking lots, and public places, there is an opportunity to surveil and track the traffic while monitoring the road, bringing forward a smart city perspective [3]. Images and/or videos of vehicles that are captured through surveillance provide a plethora of opportunities through scene understanding, object detection, recognition, and segmentation using automated approaches such as image processing, machine learning, and deep learning [4,5,6]. Further subtasks are performed from these approaches, such as re-identification [7], tracking, and similarity matching [8,9,10]. Transfer learning has been widely utilized for its computing efficiency using existing pre-trained models for video surveillance [11]. The requirement for robust vehicle identification lies in the need for public safety and security. Accuracy and real-time requirements are the prime concerns for this application. Privacy is another element that is a requirement in public surveillance.

To achieve this objective, surveillance studies of vehicles have used machine learning and deep learning models applied to vehicle data to infer the make, model, and license plate region [12]. In each case, either the wholesome image was used for analysis or a region of interest was carved where rectangular boundaries were drawn to identify the exact location of the contextual features to categorize or re-identify [7] the vehicle at another location.

In the context of cars, the car’s make is most prominently defined by the frontal view of the car [13]. The region of interest can be extracted from this view to identify the car’s make and model. This enables a better representation of the uniqueness of the car. Further, the license plate can also be extracted, which can be fed to an ALPR (automatic license plate recognition) system for digit recognition, enhancing the identification of the vehicle.

In computer vision, region of interest extraction has been a task accomplished by segmentation. The cropped region of interest is sometimes used as a pre-processing step for both deep learning and machine learning approaches [14]. A pre-set set of unique features from these images is extracted for the machine learning algorithm, whereas auto-feature extraction is performed by deep learning models.

The data presented for learning, being key to the performance and validity of the algorithms for a given task, requires rigorous labelling and reviewing. The images and/or videos captured are those of varied illumination, background, and views, making the data challenging to learn [15]. With the region of interest extracted and labelled with key significant features, there can be an improvement in learning, as seen in many state-of-the-art methods concerning segmentation and classification [16].

**Figure 1:**
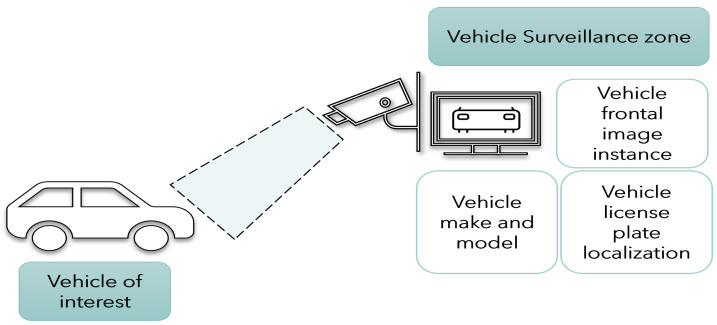
The process of vehicle surveillance at the camera for make and model classification and license plate localization.

**Figure 2:**
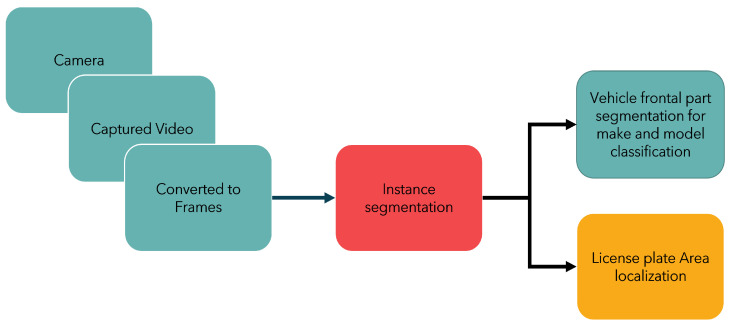
Vehicle instance segmentation technique for license plate localization along with make and model classification.

Instance segmentation is a task used in tracking. A region of interest (ROI) segmented with each instance of that specific segment can be marked and identified, enabling not just detection but also tracking of individual objects in a scene [17]. In this context, utilized here is a multi-class instance segmentation for vehicle make and model recognition clubbed with license plate recognition, as presented in Figure 1 and Figure 2. Typical make and model identification techniques need a multi-step approach for vehicle frontal-part segmentation and then classification of the detected vehicle. In this paper, a segmentation network is proposed that not only identifies the vehicle make and model under varying conditions but also precedes it by segmenting the significant frontal part of the car as a single instance, which safeguards privacy and is essential for individual unique identification and tracking. This paper presents an unique region-of-interest-labelled dataset for instance segmentation with polygonal annotations and vehicle make classification and license plate localization using deep learning.

Within this framework, a higher accuracy for the same task on the same dataset is achieved. The inference time for the two approaches is reduced as identification of the vehicle type and license plate is performed simultaneously. To improve the dataset for class imbalance, data augmentation is performed in different representations and is evaluated on the same dataset. This produces a robust and accurate model for identification of vehicles in traffic, security-sensitive roads and entrances to high security areas.

The contributions of this paper are as follows:

- An instance segmentation model for vehicle recognition through segmentation and classification. A single model for identifying a vehicle and identifying the make of the model with license plate.
- Achieving a higher mAP of detection with a deformed convolutional network with a small dataset augmented by the mosaic-tiling method.
- Analysis of several augmentation techniques and their effect on the recognition and detection of vehicle make identification using feature pyramid, deep residual and deformed deep residual networks.

In comparison to existing literature using the same dataset in [14], this method produced higher classification accuracy, with a 25.5% increase. Further, the inference time is reduced to milliseconds. Polygonal annotation of the frontal region of the vehicle is a novel approach leading to a high mAP of 99.67% for segmentation. Thus, when compared to the full vehicle instance segmentation using the KITTI dataset, the model achieved only 92%, as demonstrated in [18].

With vehicle make classification and license plate localization achieved through instance segmentation, the goal was to discover the ability of the deep learning model to perform on a polygonal dataset. In this regard, an ablation study was conducted on a deep learning framework by modifying its backbone to measure the accuracy and time complexity of each, through which the reliability of each approach was measured. Additionally, to evaluate the dataset for generalization and reducing the imbalance, an ablation study using various image augmentation techniques was performed. With the vehicles’ frontal part segmented, private surveillance was achieved.

## 2. Literature Review

Vehicle recognition is a widely researched area in the field of computer vision, categorizing itself in different tasks such as vehicle make and model recognition (VMMR), vehicle license plate recognition and vehicle re-identification [19,20]. Each task is performed individually or consecutively. The application of this comes with requirements of traffic regulation systems, smart city automation, public security and even non-civilian use cases [21]. In this paper, we take into consideration the requirements of a private and efficient automated vehicle make recognition system through instance segmentation.

Recent literature in this domain solves the challenges of private surveillance with dataset diversity with multiple large scale datasets containing a large number of classes [13,22]. This enhances not just privacy but also efficient vehicle recognition, with several datasets focusing on the frontal area of the car enabling more fine-grained classification. However, with similar vehicle features and diverse environments there still exists unique challenges in vehicle recognition. Changing vehicle ecosystem involving new manufacturers and new models has led to an open research domain in this field. There is a requirement, however, for segmentation datasets annotated in polygonal format capturing enhanced contextual features of a vehicle which is currently non-existent. With the aim of privacy and public security in its application, this paper utilises a dataset from [14] for instance segmentation of the frontal part of the car which includes, segmentation, detection, and classification.

Classification of vehicle make is performed using traditional rule-based approaches which are prominent in this field due to the popularity of the problem. Local and global cues have been utilized for classification in several approaches. Structural and edge-based features have also been a common pick. Further, machine learning has been performed with these features to enhance classification. With the feature extraction techniques, edge-based feature extractors, such as HOG and Harris corner detectors, have performed significantly well for detecting parts of the car such as the logo, the grille or the headlights [23]. Robust feature detectors from key points, such as SIFT and SURF, have been employed in several state-of-the-art models. In addition to these features, corner and line detectors, such as Hessian matrix and DoG (difference of gaussian), have been implemented, producing considerably a higher accuracy for a smaller number of classes [14,24]. With a larger number of classes, these models failed to produce a similar accuracy. Further, a bag-of-features or a bag-of-words approach has been implemented with feature detectors for unsupervised clustering producing a histogram of features for matching [25]. A typical feature detector algorithm accompanies a matching technique, such as hamming distance, euclidean distance, or cosine similarity, to identify similar vehicles for recognition and classification. This is further used for re-identification tasks.

Naïve Bayes [26], SVM [27], LBP [27], and KNN are common machine learning algorithms that have been used for vehicle make and model classification. CNN architecture used for vehicle make and model classification involves transfer learning on prominent pre-trained models, such as Alexnet, VGG, Resnet, and mobilenet [28]. In addition to this, modified CNN networks were introduced, such as residual squeezenet [2], which produces a higher rank-5 accuracy of 99.38%. Segmentation has been applied as a pre-processing step to remove background noise. The compound scaling approach has been employed on EfficientNet pre-trained on ImageNet for vehicle make and model classification. Unsupervised deep learning techniques such as auto-encoders have also been utilized for this purpose [27]. Apart from frontal images, recently a part-level feature extraction method where feature grouping was utilized by Lei et al. in [29] was employed to classify and recognize vehicles. This method produced an recognition accuracy of 97.7%. A genetic algorithm for feature optimization of CNN-generated features was utilized in [30]. Classification was performed using an SVM classifier. A hybrid CNN–SVM method was performed which produced an accuracy of 99.71%; however, this method failed to present license plate localization or region of interest segmentation.

Segmenting the region of interest achieves better recognition and private surveillance. In vehicle identification, segmentation approaches are often used to remove the background and extract the vehicle to classifying it [27,31]. In a real-time use case, cropped images should be generated from an image that will later be used for part detection. Almost all approaches necessitate an extra step for vehicle detection, which adds to the time complexity. As a result, a one-step approach for vehicle identification is required. License plate detection adds to the vehicle’s unique features, which are then added to the identification system for re-identification of the vehicle’s unique ID tagging. As a result, a robust model is required that can detect the region of interest, identifying each instance of the vehicle’s make.

We consider this challenge in this paper and propose instance segmentation for vehicle identification via segmentation and classification. A two-stage approach for feature extraction using FPN (a feature pyramid network produced by multi-scale feature extraction) [32] and classification using maskRCNN [33] is utilized in this paper. Further experimentation is performed on a modified CNN to improve the performance of the network. Image augmentation techniques are explored for the purpose of improving the existing dataset.

## 3. Methods

Convolutional neural networks have been key in computer vision applications. They are the most commonly used type of artificial neural networks. Convolutional operations applied to neural networks enable better feature extraction and classification [34]. Convolutional neural networks have evolved based on the requirements of accuracy, generalization and optimization problems. The need for generalization and domain adaptation has led to a rise in several large-scale models trained on large-scale data. Large-scale data is trained on these networks which can be further adapted to other applications. Examples of convolutional neural networks include, Alex net [35], Lenet [36], Resnet [37], Google-net [38], Squeeze-net [39], and so on. In this paper, we utilize Resnet, a deep residual network consisting of multiple CNN layers. Resnet extracts deep features and with its residual skip connections, the network is efficient in solving the vanishing gradient problem [37].

Convolutional neural networks comprise of four key features which include weight sharing, local connection, pooling and a large number of layers [40]. The layers include the convolutional layer that performs the convolutional operation on small local patches of the input where a given input *x* with a filter *f* produces a feature map of *x*. The convolution operation for the whole image is computed by the following, as shown in Equation (1).

(1) $$Y\text{\_}n=\sum_{k=0}^{N-1}\left(x\text{\_}k\right)\left(f\left(n-k\right)\right)$$

where *x*, *f*, and *N* are the input image, filter, and the number of elements in *x*, respectively. The output vector is represented by *Y*.

This is followed by activation functions such as tanh, sigmoid and ReLU [41]. The activation functions introduce non-linearity into the network. The sub-sampling layer that are the pooling layers reduce the feature map resolution leading to reduced complexity and parameters. The extracted features are mapped to the labels in the fully connected layer. All the neurons are transformed into a 1D format [42]. The outputs of the convolutional and sampling layers are mapped to each of the neurons producing a fully connected layer. The fully connected layer is spatially aware extracting locational features as well as producing high-level complex features. The result of this is linked to the output layer which produces the output using a thresholding process. A final dense layer is sometimes used with the same number of neurons as classes in the case of multi-class classification. A softmax activation function maps all the dense layer outputs to a vector producing a probability for each class.

The accuracy of this prediction is measured by its loss function where the result is compared to the ground truth or labelled data. A commonly used loss function is the categorical cross-entropy loss computed as *L*, as shown in Equation (2).

(2) $$L=-\sum_{i=1}^{N}\left(y_{i}\cdot log\left(y\;\hat{i}\right)\right)$$

This setup is trained through a backpropagation technique. Hyperparameters such as the learning rate, regularization and momentum parameters are set before the training process and adjusted according to the brute-force technique. Evolutionary algorithms are further used to automate hyperparameter tuning. During the backpropagation technique, the biases and weights are updated. The loss function *L*, as shown in Equation (2), is required to be of minimum order to produce an accurate model. For this purpose, parameters, such as kernels (filters), and biases are optimized to achieve the minimum loss. The weights and biases are updated in each network and the feed-forward process is iterated with the updated weights. The model converges at the least loss.

Deep residual networks are utilized as the backbone. Deep residual networks are large networks with skip connections that carry knowledge. The methodology utilized in this framework performs instance segmentation using a CNN. Instance segmentation enables detection and delineation of each object in a given image or video. Each instance of an object is tagged with an ID enabling unique detection of every object in the scene.

### 3.1. Deformable Convolution

With all the advantages of the convolutional neural network, the geometric structures of its building modules are fixed. Augmentation is used for transforming images as a pre-processing step in most convolutional neural networks. Thus, these transformations, such as rotation and orientation, are fixed by modifying the training data. The structure of the filters in the kernel are also fixed in a rectangular window. Pooling mechanisms produce the same size kernels to reduce special resolution and thus the objects in the same receptive field are convoluted and presented to the activation function. Therefore, only objects in that scale are identified. Deformable convolution enhances geometric transformation and scaling by introducing a 2D offset to the grid sampling locations and thereby the convolution operation offsets from its fixed receptive location to a deformed receptive field. Adding the offset automatically augments the spatial sampling locations. The offsets are added after the convolutional operation.

Further, to enhance detection at lower levels image pyramids are computed to build a feature pyramid network. The object or segmentation area is scaled over different position levels in the pyramid. Proportionally sized feature maps at multiple levels are generated from a single input. Then, cross-scale correlation is generated at each block to generate a fusion of these features. FPNs are used with CNNs as a generic solution to build feature maps. A bottom-up or top-down approach is then used to produce a feature map. In terms of deep residual networks, the feature activation outputs are produced at each stages’ last residual block.

**Figure 3:**
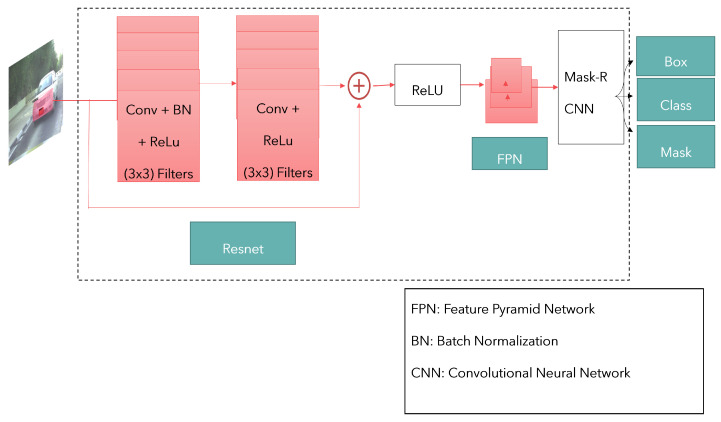
The maskRCNN with FPN architecture.

In this paper, we implement a maskRCNN with a Resnet backbone and a FPN. The use of this network is justified due to its accuracy in object detection and segmentation when it is pre-trained on several large datasets which have superior performance over other models. However, the complexity of the model causes the time complexity to increase. Therefore, we further measure the trade-off accuracy vs. time enabling the evaluation of a real-time use case. Figure 3 depicts the architecture of the maskRCNN with FPN used for instance segmentation. The maskRCNN is a region-based CNN that performs object detection and classification with mask generation. The object detection is performed on a region of interest and then evaluated. A multi-task loss is sampled from the region of interest as a total classification loss, and object detection loss is the bounding box loss and mask loss. Complex hierarchical features are extracted from images. With extensive evaluation, the models are susceptible to overfitting; therefore, regularization techniques are required to improve this overfitting.

### 3.2. Data Augmentation

**Figure 4:**
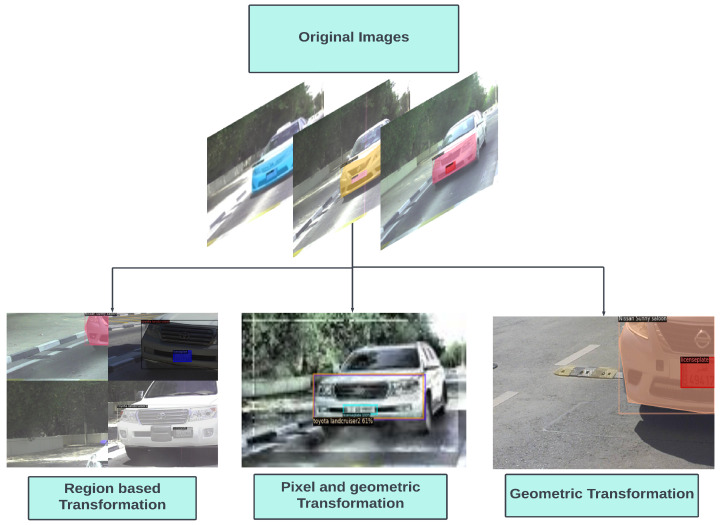
Augmentation techniques.

**Figure 5:**
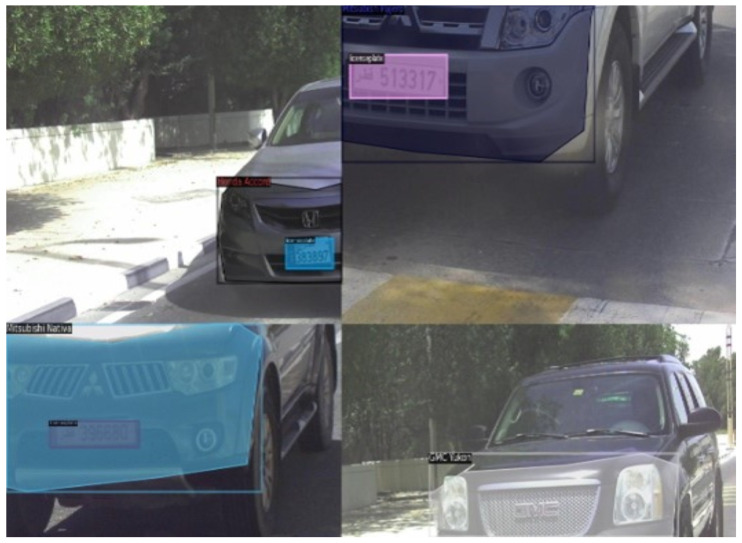
Mosaic-tiled augmentation.

Augmentation techniques are often applied to reduce overfitting, this includes image transformation, such as scaling, translation, rotation and random flipping. This not only increases the data size but also provides a diversity of representation. The augmentation techniques can be divided into pixel-level data, region-based and geometric data augmentation. Pixel-based augmentation techniques include changes in pixel values. Adding contrast, brightness and colour changes the pixel intensity of the image. Regional augmentation includes creating masks of the required region. Motion blur and cut-out are common techniques used for region-based augmentation. Geometric transformations are also applied to data including flipping, reflection, rotation, cropping, etc. In this paper, we set up the data to augment at different levels that include geometric transformation and region-based transformation, as seen in Figure 4. This not only enhances the dataset but also improves dataset diversity. One particular approach used in this model is the mosaic-tiling method proposed in [43], where different training images, in this case four, are taken in different context and stitched into one image to create a sort of mosaic tiling. Random cropping is performed on the image to reduce it to the original training image size. Figure 5 is an illustration of the mosaic-tiled images of the dataset. Thus, a baseline method is used for instance segmentation and then modified and evaluated in terms of data augmentation, different feature extractors, and deformed convolution to identify the effect of each and chose the optimum configuration for vehicle instance segmentation.

## 4. Experimental Setup

The setup of this network involves three layers. The vehicle with a mask is fed in as the training data. The data is separately augmented in three formats based on geometric and pixel-based augmentation. The transformed data is taken as the testing data and then trained on a maskRCNN-FPN network. Further, an experiment was performed on the maskRCNN-FPN by deforming the convolutional layers. Resnet-101 and 50 were used as feature extractor backbones to perform baseline assessments on the dataset. All experiments were ran with a learning rate of 0.001 and 5000 iterations. The setup is as shown in Figure 3. The experiment was performed on an Intel(R) Xeon(R) CPU @ 2.30 GHz using GPU virtual instance on an Ubuntu machine (Asus, China).

### 4.1. Dataset

**Figure 6:**
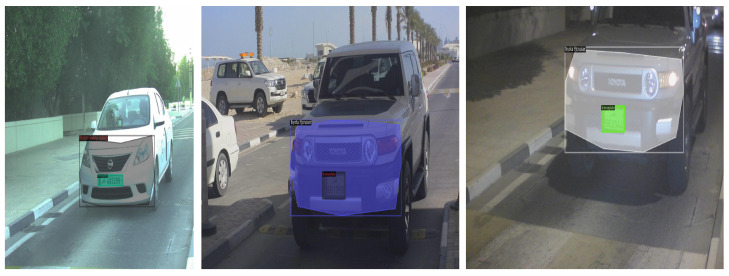
Dataset images.

An existing dataset [14] was modified for instance segmentation by creating polygonal bounding boxes of the frontal part of the vehicle to capture the frontal dashboard and the curvature of the vehicle. The dataset contains 12 vehicles makes taken from different camera exposures during extremely sunny weather to evening sunset. The dataset is imbalanced and therefore augmentation was performed to improve the data count. In addition, the license plate was treated as a single class having a rectangular bounding box. Figure 6 shows the vehicle samples with their annotations. A total of 225 images were split for training, testing and validation with 157 images for training, 44 images for validation, and 24 images for testing (a 70:20:10 ratio from the original format). This split was utilized to match the split of the reference paper [14]. The classes were very imbalanced and required further augmentation, performed as per the methodology stated earlier. The image below displays the class distribution of the dataset. This dataset contains vehicles that belong to the middle-eastern region, specifically Qatar.

**Figure 7:**
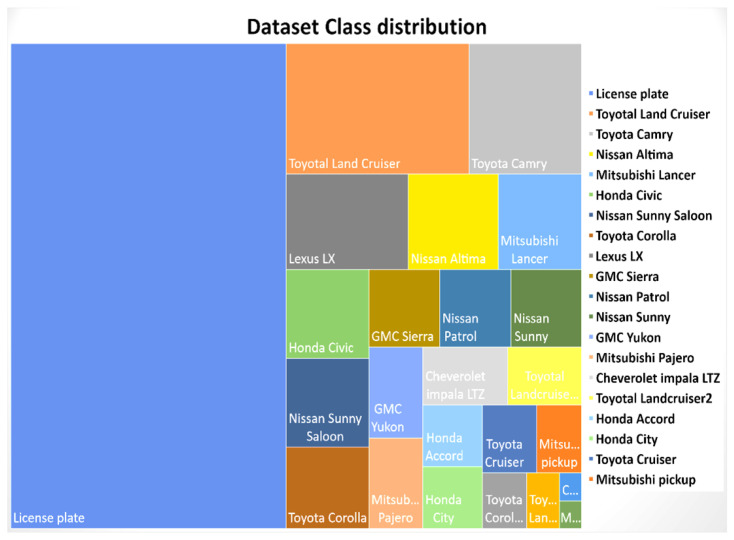
Dataset distribution.

The experiments were conducted by augmenting the dataset to mimic different camera orientations and noise parameters. An evaluation of both the original dataset and partly augmented dataset was performed. The augmentation parameters included in the pixel- and geometric-based augmentation include exposure and resizing with auto-orientation, noise, and rotation. Further, patch-based augmentation was performed which is a type of geometric augmentation. The third type of augmentation was the mosaic-tiled approach. The dataset with annotation is available at [44]. Figure 5 is an example of data augmentation performed on the dataset and Figure 7 shows the distribution of classes across the whole dataset.

### 4.2. Performance Metrics

To calculate the average accuracy, precision and recall must be computed for each image. TP (true positive), FP (false positive), FN (false negative) and TN (true negative) are the metrics used for precision and recall. Equations (3)–(5) were used to compute the accuracy, precision and recall, respectively.

(3) $$Accuracy=\frac{Correctpred.}{Totalpred.}=\frac{\left(TP+TN\right)}{\left(TP+TN+FP+FN\right)}$$

(4) $$Precision=\frac{Truepositive}{Predictedpositive}=\frac{1TP}{\left(TP+FP\right)}$$

(5) $$Recall=\frac{Truepositive}{Actualpositive}=\frac{TP}{\left(TP+FN\right)}$$

**mAP: mean average precision per class** Average precision (AP) measures how well the model classifies each class, while mean average precision (mAP) measures how well the model classifies the whole given test dataset. It is a measure of identification accuracy. It evaluates the performance of the model by averaging the precision under the IoU (intersection over union) with a threshold from 0.50 to 0.95, with a step of 0.05. The AP is calculated for each point within the threshold. For different queries, the evaluation metrics are APS, APM, APL, AP50, AP75, and mAP. Subscripts “S”, “M”, and “L” refer to “small,” “medium,” and “large,” respectively. Subscripts “50” and “75” represent the IoU thresholds of 0.5 and 0.75, respectively. The mAP is the mean AP for each experiment.

**Inference time:** The inference time is measured by the time taken to classify and generate a mask for a single input. In the context of this approach, the inference time will be taken to classify and generate masks for a single frame of a video.

## 5. Results and Discussion

Several experiments were conducted for different augmentation methods on the dataset. The Resnet-50 backbone was used for the deformable receptive field-based maskRCNN. With a batch size of two, the experiments ran for 1000 iterations and used a pre-trained Resnet backbone on the COCO dataset. The evaluation was performed using the COCO trainer module. The results without segmentation are listed in Table 1 and the ablation study based on different backbones and feature extractions is tabulated in Table 2 with the original dataset size, resolution, and clarity.

For a varied analysis different baselines were experimented on for the purpose of evaluation and identifying the trade-off in the reliability and accuracy of an instance segmentation approach for the purpose of vehicle recognition. The maskRCNN was used as a baseline with a Resnet-50 backbone and FPN. Further, the maskRCNN was modelled with a Resnet-101 backbone with FPN. The original dataset was augmented with multiple methods to improve the dataset description. The results of experiments with the original dataset is displayed in Table 1 and Table 2. The Table 1 describes the classification accuracy of the maskRCNN with the instance segmentation accuracy and mean average precision metric. The execution time for inference of a single image from the test set is also presented. The Resnet-50 backbone with a base RCNN without the FPN produces a high mAP of 99.670%.

**Figure 8:**
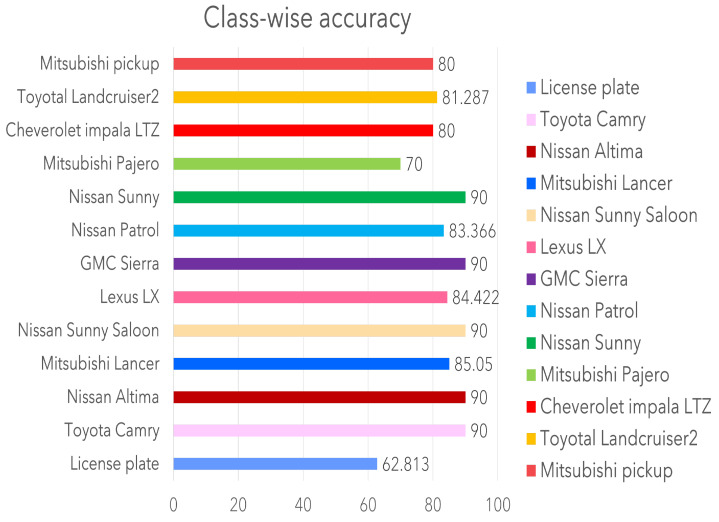
Class-wise accuracy based on the maskRCNN–Resnet-50 results.

Although the Resnet-50 backbone with the FPN is hypothesized to produce a higher accuracy, it lags by 1% but produces a faster inference, 174 ms faster than the base-RCNN. Further experiments on the CNN module with a deformed convolutional operation the accuracy dropped to 90%, significantly less than expected. This could be due to the added complexity and generalization of the network. It should be noted that the models are inferred on a test set with imbalanced data and thus are not reliable for certain classes. With class-wise precision, it should be noted that the largest class, license plate detection has the poorest accuracy. This poor performance may be because the license plate covers a small area and is similar to other rectangular shapes. Class-wise performance is depicted in Figure 8.

**Figure 9:**
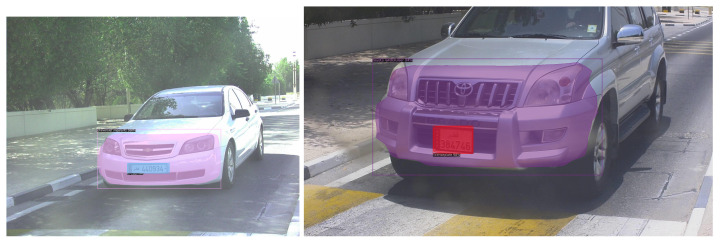
Resultant images of segmentation and classification using the maskRCNN with the Resnet-50 backbone.

The test data is either over- or under-represented and thus has to be balanced for reliable results. Thus, multiple augmentation techniques were performed to improve data representation. Three types of augmentation approaches were utilized for this task. Table 3 describes the results and the approaches used. Large and small networks were tested to evaluate the impact of augmentation on data size and model accuracy. The table describes the results of each augmentation type on the baseline models. The inference from the table is clear that mosaic augmentation performs considerably better than any other augmentation type. However, it fails to surpass images with the same resolution. The patch-based augmentation has a much lower inference than expected even though the number of images increase. This could be because of class-empty patches in the dataset as each class is represented once in the original image. With per class evaluation, each class performed well in every model achieving an average of around 80%. However, license plate detection was a challenge for many of the models with 62.813% as the highest mAP compared to all the other networks. The number of images did not have an impact on the performance of this class, which may be attributed to the reduced size of the license plate and its location in images with respect to models such as Lexus. Figure 8 shows the per class result of the maskRCNN with the Resnet-50 backbone with the highest accuracy for the Toyota corolla compared with other classes. Figure 9 shows a resultant image of segmentation where the frontal area of the vehicle is segmented and the make identified with license plate localization.

### Benchmarking

Bench-marking existing literature, the classification accuracy using the existing dataset is given in Table 4. The table shows an significant increase in accuracy compared to traditional methods using SIFT and DoG. The notable change in the model complexity and technique produce the difference in these parameters. Distinct features are globally extracted compared to the constant local feature points in the dataset. Comparing existing results on the same dataset, a considerable increase in recognition accuracy was achieved on the test data. Although it stands out from other models, it can be seen from Figure 8 that classes with a low number of images were not part of the test data. Therefore, an imbalance is noted.

## 6. Conclusions

Instance segmentation of a vehicle’s frontal region is an effective tool for vehicle classification and identification. Existing techniques require multiple steps to identify a vehicle, segment and then identify the make and model from this data using multiple algorithms or separately trained networks for each task. In this approach all tasks were achieved with one model. Time complexity was measured and the approach that exhibited the lowest execution time (136 ms) was the maskRCNN with the Resnet-50 and FPN. With an enhanced dataset with instance segmentation and further data augmentation of the performance an overall evaluation is presented. However, new models of vehicles need to be added to the data to balance the dataset for further improvement. Further, evaluation is required for light weight models, such as the centre mask [45] model which is an anchor-free approach that can further improve the inference time. The instance produced from this model could be further used for re-identification as each unique instance is created for each vehicle per model. Privacy is further advanced with processing proposed in a blockchain network rather than a centralized storage as each instance of the frontal part of the vehicle can be saved rather than the whole image itself. Thus, securing the privacy and reliability of the automatic vehicle recognition system is achieved.

## Figures and Tables

**Table 1:** Classification accuracy and detection accuracy using mAP with latency.

Model	Lr	Fast_rcnn/cls_accuracy	mAP	Time
MaskRCNN + RESNET-50 + FPN	3×	0.992	98.772	136 ms
MaskRCNN + RESNET-101	3×	0.996	88.219	310 ms
MaskRCNN + RESNET-50	1×	0.992	99.670	316 ms
MaskRCNN + RESNET-50 + FPN (DCONV)	1×	0.984375	90.747	161.81 ms

**Table 2:** Ablation study with different backbones and deformable convolution.

Model	Model	AP	AP50	AP75
MaskRCNN-DCONV	RESNET-50 + FPN	79.648	96.337	94.350
MaskRCNN-DCONV	RESNET-50 + FPN	74.185	90.747	89.121
MaskRCNN	RESNET-50 + FPN	80.213	98.772	95.950
MaskRCNN	RESNET-101	73.621	88.219	86.265
MaskRCNN	RESNET-50	80.206	99.670	98.730

**Table 3:** Ablation study on data augmentation.

Aug. Type	(Train-Test-Split)	Model	Backbone	AP	AP50	AP75
Resize+expo.	471-44-24	MaskRCNN-DCONV	Resnet-50 + FPN	65.748	81.708	77.517
		MaskRCNN	Resnet-101	70.989	88.633	85.148
		MaskRCNN	Resnet-50	59.502	85.189	67.677
Full Aug.	460-44-24	MaskRCNN-DCONV	Resnet-50 + FPN	66.780	83.101	75.029
		MaskRCNN	Resnet-101	49.585	66.776	58.586
		MaskRCNN	Resnet-50	60.163	77.906	73.954
Patch input	628-176-96	MaskRCNN-DCONV	Resnet-50 + FPN	52.475	74.535	64.246
		MaskRCNN	Resnet-101	71.569	88.176	84.842
		MaskRCNN	Resnet-50	52.186	74.393	59.095
Mosaic Based	471-44-24	MaskRCNN-DCONV	Resnet-50 + FPN	87.698	99.406	98.900
		MaskRCNN	Resnet-101	83.933	99.568	99.103
		MaskRCNN	Resnet-50	82.463	99.637	98.121

**Table 4:** Comparison with the existing literature.

Reference	Model	Classification Accuracy
[14]	SIFT + DoG	74.63%
Ours	MaskRCNN+ FPN + Resnet-50	99.2%

## Data Availability

Part of the experimental and annotated data are available at https://drive.google.com/drive/folders/1zqR1s9YiTxAfjfF213WbiH3Xc-SHPIPs?usp=sharing (accessed on 1 December 2022).

## Abbreviations

The following abbreviations are used in this manuscript:

CNN	Convolutional neural network
D-CONV	Deformed convolution
TL	Transfer learning
RESNET	Residual network
FPN	Feature pyramidal network
